# Silicon microgrooves for contact guidance of human aortic endothelial cells

**DOI:** 10.3762/bjnano.8.72

**Published:** 2017-03-22

**Authors:** Sara Fernández-Castillejo, Pilar Formentín, Úrsula Catalán, Josep Pallarès, Lluís F Marsal, Rosa Solà

**Affiliations:** 1 Unit of Lipids and Atherosclerosis Research, Department of Medicine and Surgery, Universitat Rovira I Virgili, Sant Llorenç 21, 43201 Reus, Tarragona, Spain; 2 Nano-electronic and Photonic Systems, Departament d’Enginyeria Electrònica, Elèctrica i Automàtica, Universitat Rovira I Virgili, Països Catalans 26, 43007 Tarragona, Spain

**Keywords:** cell morphology, contact guidance, microgrooves, silicon, human aortic endothelial cells (HAECs)

## Abstract

**Background:** Micro- and nanoscale substrates have been fabricated in order to study the influence of the topography on the cellular response. The aim of this work was to prepare different collagen-coated silicon substrates displaying grooves and ridges to mimic the aligned and elongated endothelium found in linear vessels, and to use them as substrates to study cell growth and behaviour.

**Results:** The influence of groove-shaped substrates on cell adhesion, morphology and proliferation were assessed, by comparing them to flat silicon substrates, used as control condition. Using human aortic endothelial cells, microscopy images demonstrate that the cellular response is different depending on the silicon surface, when it comes to cell adhesion, morphology (alignment, circularity and filopodia presence) and proliferation. Moreover, these structures exerted no cytotoxic effect.

**Conclusion:** The results suggest that topographical patterning influences cell response. Silicon groove substrates can be used in developing medical devices with microscale features to mimic the endothelium in lineal vessels.

## Introduction

Micro- and nanostructured materials for medical devices have demonstrated that surface topography as well as surface chemistry influence cellular behaviour such as adhesion, migration and proliferation [1–6]. It is important to understand and control cell behaviour by topography in order to modulate the functions of the cells. Cells react to topographic stimuli through a process known as mechanotransduction. Reactions of cells to topography are different in the nanometre and micrometre range. Different patterns cause differences in migration or adhesion. Moreover, morphology and orientation of the cells are also influenced by the contact guidance, also known as topographic guidance [7]. This concept refers to the affinity of the cell to be elongated and guided by the shape of the surface. The response of the cells to these topographical cues and the concept of contact guidance have been described previously for a wide variety of cells such as neuronal, epithelial or endothelial cells [8–10]. In particular, adhesion and orientation of endothelial cells on different surfaces can be controlled by combining surface chemical treatment and topography mimicking the elongated endothelium characteristic of the blood vessels [11]. A broad range of techniques and materials have been employed to fabricate well-defined topographical and chemical cues to assess cell micropatterning [12–16]. Some of these approaches are based on photolithography and reactive ion etching that in some cases are followed by anisotropic etching [17].

A simple and effective geometry previously described, involves line-shaped features that consist of repeated ridges and grooves pattern. Surfaces with these geometries have been used to demonstrate the influence on cell adhesion, alignment and organization [18–21]. Differences in cytoskeleton elongation have been demonstrated between cells elongated on these surfaces and cells cultured on unpatterned surfaces [22–26].

Silicon is a useful material to create surfaces containing a wide variety of arrayed features. The architecture and the intrinsic properties of silicon, such as its surface stability and solvent compatibility, are important features for its application in biotechnology and biomedicine [27–28]. Silicon dioxide is nontoxic and biocompatible, and based on these features it has been proposed as material for drug delivery in cell culture models and for tissue engineering [29]. In addition, silicon offers a flexible surface chemistry that allows one to link bioconjugators such as collagen [30]. Collagen is an attractive tool for biomedical applications as it is the most abundant protein in mammalian tissues [31]. We have recently reported the development of collagen-coated silicon-based microstructures, classified as nanoporous, macroporous and micropillars, to study the effect of topography on the behaviour of endothelial cells. Collagen was found to stimulate cell adhesion and promote an enhanced cell attachment [32–33].

Herein, to mimic the elongated endothelium in natural lineal vessels, human aortic endothelial cells (HAECs) have been cultured on grooved silicon substrates coated with collagen. The HAEC cell line is one of most commonly used models in the study of the endothelial dysfunction and its capacity to adhere to the substrate and to produce cell adhesion molecules makes them a good tool for screening new cardiovascular therapies [34].

The aim of this work was to prepare different collagen-coated silicon substrates with grooves by photolithography, and to study the cell behaviour on such structures compared with that on flat silicon substrates, used as control.

## Results and Discussion

### Fabrication of grooved silicon substrates

**Figure 1:**
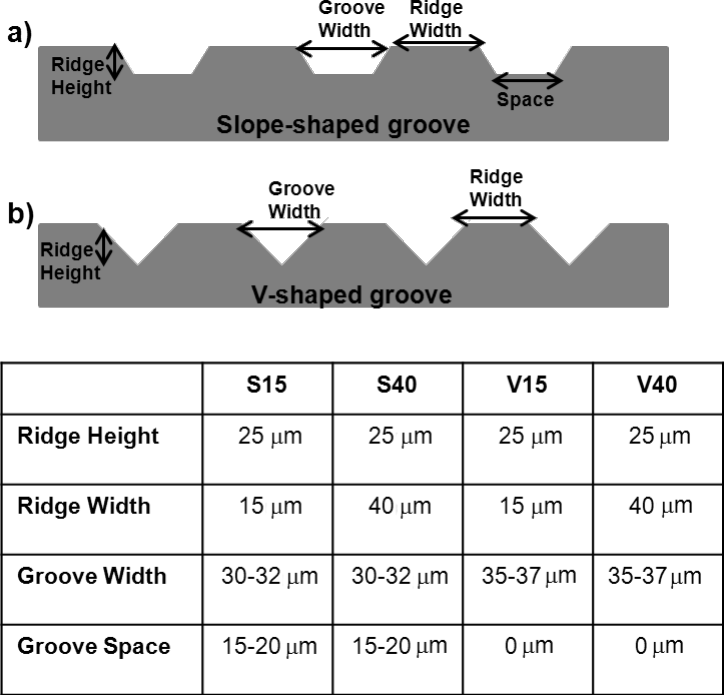
Side view and features of the silicon substrates produced. a) Slope-shaped grooves and b) V-shaped grooves.

**Figure 2:**
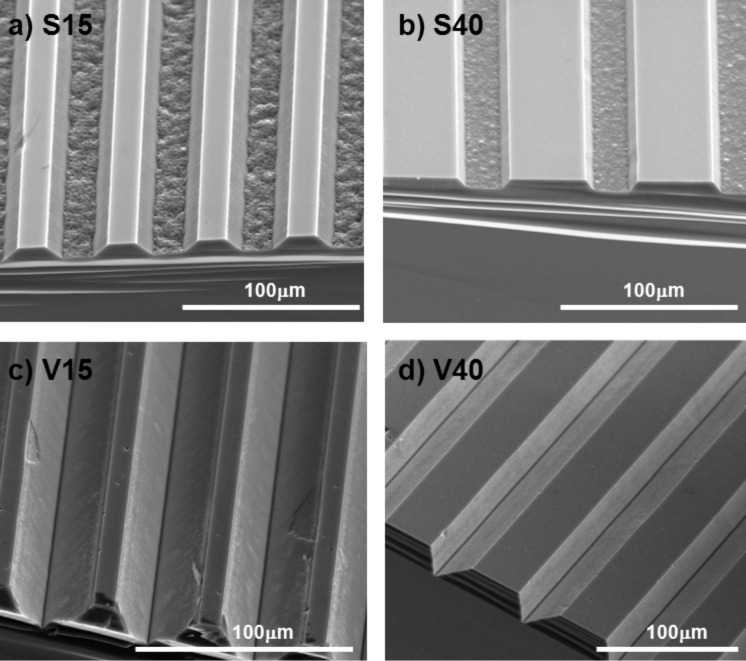
ESEM images of grooved silicon substrates. a,b) Slope-shaped grooves and c,d) V-shaped grooves.

To study the cellular response on surfaces with different geometry, different grooved substrates were produced in silicon wafers using standard photolithography and wet etching techniques [35–36]. The etching time in tetramethylammonium hydroxide (TMAH) was varied in order to generate two types of grooves (V-shaped and slope-shaped grooves). Figure 1 schematises the features of the silicon substrates produced, while Figure 2 shows representative images of the topography of such structures, analysed using environmental scanning electron microscopy (ESEM).

As shown in these figures, four structures were produced differing in height and width of the ridges and grooves: slope-shaped grooves with a ridge groove of 15 μm (S15) or 40 μm (S40) and V-shaped grooves with a ridge groove of 15 μm (V15) or 40 μm (V40). Flat and micropatterned silicon substrates were bio-activated to promote cell adhesion and surface stability following the 3-amimoptopyl triethoxylane (APTES)–glutaraldehyde (GTA)–collagen sequence as described in Experimental section.

### Cytotoxicity of silicon substrates

**Figure 3:**
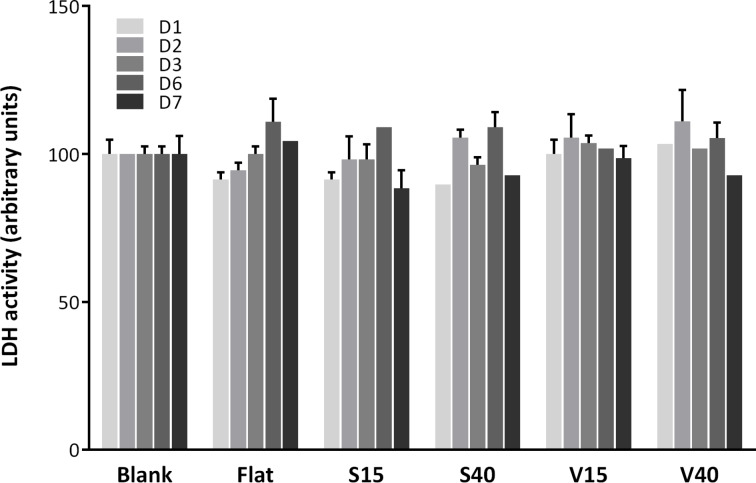
Cytotoxicity observed after D1–D7 of cells incubation on a regular well-plate (blank condition), on flat substrate (Flat) or on different patterned substrates (S15, S40, V15 and V40). No statistical differences were found in any condition tested.

Cytotoxicity was assessed by measuring LDH activity 24 h, 2 days, 3 days, 6 days and 7 days (D1–D7) after incubating the silicon substrates with human aortic endothelial cells (HAECs). Blank control values (cells seeded in the absence of any silicon substrates) were set at 100% and the other conditions were calculated in relation to this reference value. As shown in Figure 3, no cytotoxicity was observed as no statistically significant changes were observed.

### Cell adhesion

The adhesion of HAECs on flat and grooved silicon substrates functionalized with collagen was assessed with ESEM and confocal microscopy after two days of culture. The results revealed that the number of adhered cells on groove substrates tended to be different to that on flat substrate.

**Figure 4:**
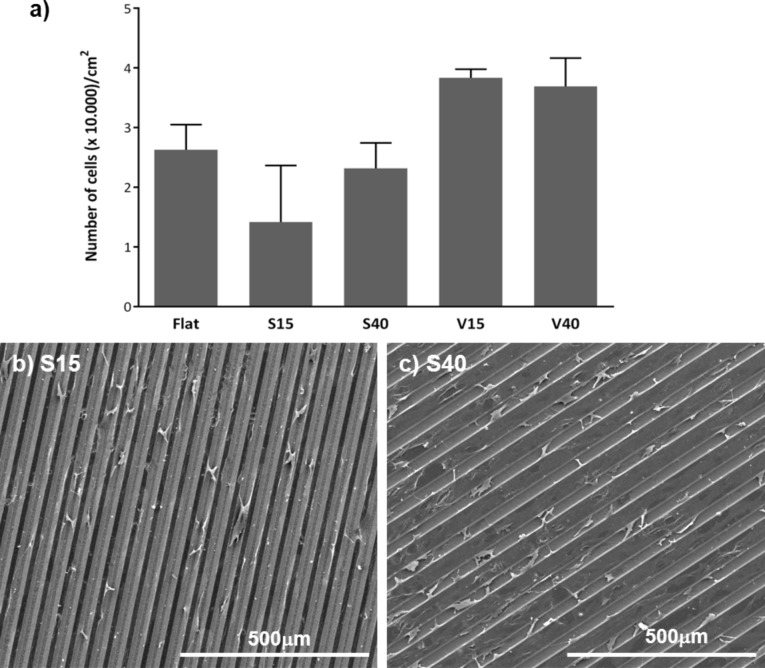
a) Attachment of HAECs after 2 days of culture on different silicon substrates. b,c) ESEM images of SL groove silicon samples after 2 days of incubation. No statistical differences were found under any condition tested.

Compared to the flat substrate, the number of cells adhered was lower in slope-shaped grooves and higher in V-shaped grooves irrespective of the ridge width (Figure 4a). Moreover, confocal microscopy images provide also information on the location of cells in the substrates. HAECs exhibit a preference to adhere to the groove surface when the ridge width is 15 μm in slope-shaped and in V-shaped structures. When the ridge width is bigger (40 μm), cells are also attached to the ridges, thereby covering the entire surface (Figure 4b,c). These data confirm that the topography modifies cell tendency to adhere, although these differences were not found to be statistical significant.

### Cell morphology

**Figure 5:**
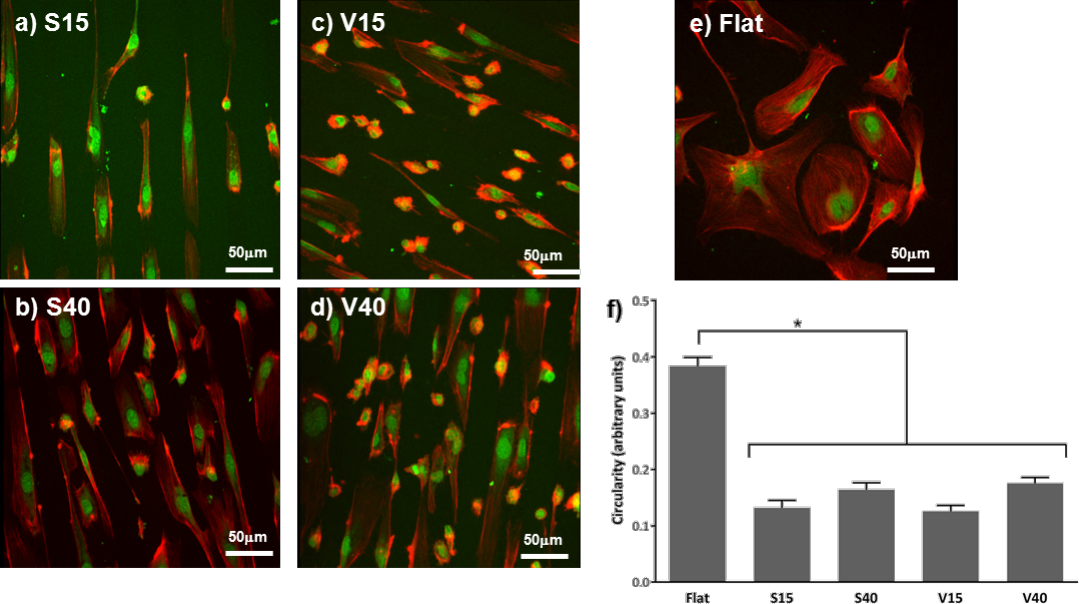
Morphology assessment. Confocal images of HAECs cultured for 2 days on a) S15 grooved, b) S40 grooved, c) V15 grooved, d) V40 grooved and e) flat silicon substrates. f) Circularity of HAECs cultured for 2 days on flat and on patterned silicon substrates (*p < 0.05).

Cell morphology was defined as the combination of circularity, alignment to the substrate structures and presence of filopodia. Figure 5a–d shows the confocal images demonstrating the contact-guidance effect of the microgrooves on cells cultured on these substrates for 2 days.

Cells cultured on flat silicon substrates exhibited a random orientation, while HAECs seeded onto grooved surfaces were elongated and aligned in the direction of the grooves. These differences in cell morphology were quantified by assessing cell circularity. As revealed in Figure 5f, reduced circularity of the cells cultured on the patterned substrates were observed compared to that on flat substrate. These differences reached a statistical significance (*p* < 0.05). That is to say, HAECs were more elongated on grooved than on flat silicon surfaces, where the cells showed a more flattened and spread morphology. Moreover, no statistical differences were observed in the circularity of cells cultured on the different patterned structures assessed.

**Figure 6:**
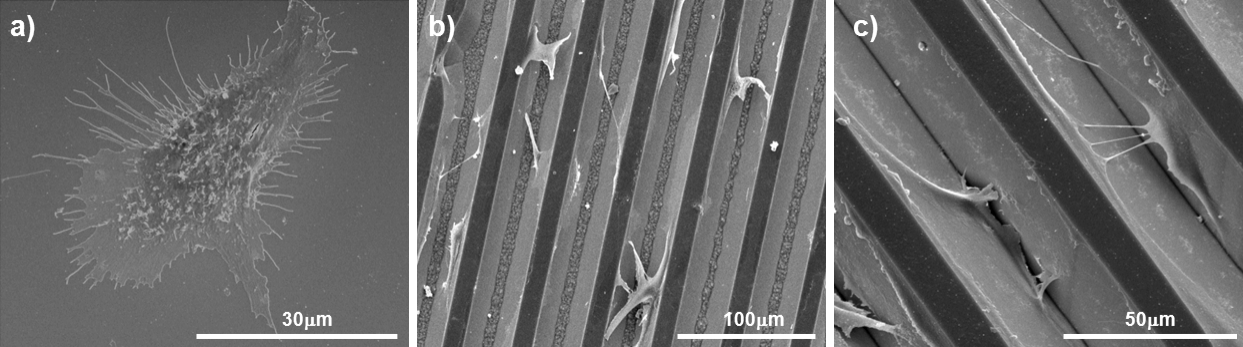
ESEM images of HAECs cultured for 2 days on a) flat, b) slope-shaped grooves and c) V-shaped grooves.

Moreover, cells cultured on unpatterned substrates presented a large number of filopodia extending from the central part of the cell as depicted in Figure 6a. Filopodia were also observed in cells cultured on patterned structures, but to a lesser degree (Figure 6b,c). When cells were incubated on the grooved substrates for 7 days, filopodia were not observed and the cell spreading seems lower than after 2 days of incubation (data not shown). Taking all these data together, it seems safe to assure that the presence of microstructure affects cell morphology, irrespective of the patterned employed.

### Cell proliferation

**Figure 7:**
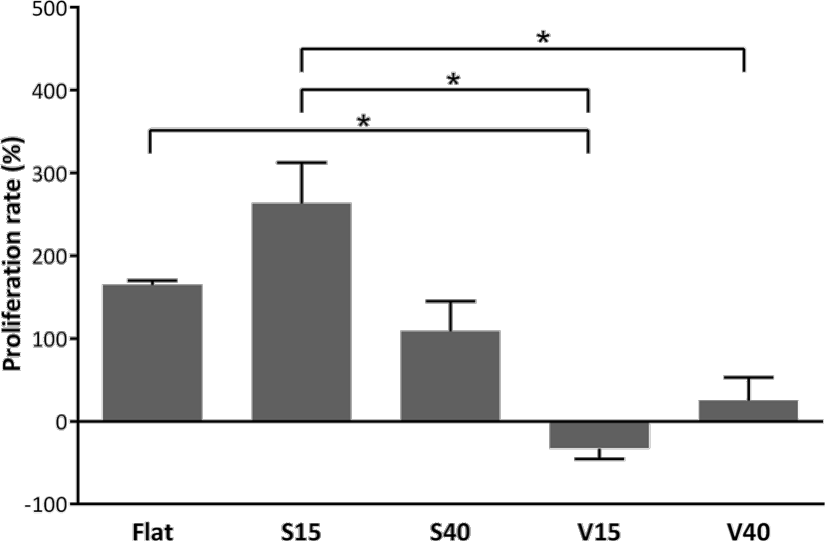
Proliferation of HAECs on flat and microstructured silicon substrates (*p < 0.05).

The effect of surface microstructure on the proliferation of HAECs was studied on day 2 and day 7. As shown in Figure 7, the number of cells cultured on flat substrates for 7 days increased by 65% compared to day 2, and this proliferation rate was statistically different to those observed in cells cultured on patterned substrates (*p* < 0.05). On the one hand, cells cultured on V15 presented no proliferation. On the other hand, cells proliferated when cultured on S15, S40 and V40 and this proliferation was different accordingly to the patterning. That is to say, proliferation was greater in S15 and lower in S40 and V40, when compared to flat substrates. This decreased proliferation observed on some micrograting has been previously reported in the literature, where it is suggested that the decrease of proliferation could be due to the decrease in cell spreading [37].

## Conclusion

In this work, four different grooved silicon substrates coated with collagen were fabricated and employed to evaluate the effect of the topography on cell adhesion, morphology (alignment, circularity and filopodia formation) and proliferation of endothelial cells. The data obtained in the present work confirms the hypothesis that these collagen-coated silicon structures consisting of repeated ridges and grooves patterning modify the behaviour of endothelial cells.

Microscopy analysis revealed differences between flat silicon and patterned substrates. Firstly, the number of attached cells was higher when cells were cultured on V-shaped substrates compared to the culture on slope-shaped substrates. Secondly, morphology was also found to be modified by the substrate patterning when compared to flat substrate. However, no significant differences were found between the four patterned structures. In this sense, endothelial cells showed significant alignment in the direction of the groove pattern, accordingly to the concept of contact guidance, and circularity and filopodia were reduced on patterned substrates when compared to flat substrates. Lastly, cell proliferation was found to be lower on patterned substrates, surely because of the aforementioned decrease in cell spreading.

Taking all the previously mentioned data, the present work provides evidence of the influence of the silicon surface topography on the cell behaviour. The use of such substrates may be a useful tool for the development of three-dimensional medical devices with microscale features.

## Experimental

### Fabrication of grooved silicon substrates

Groove samples were prepared on p-type silicon(100) wafers with a resistivity of 1–5 Ω·cm. The wafers were thermally oxidized at 1000 °C for 15 min in order to grow a thin SiO_2_ layer that will act as a mask in the anisotropic alkaline etch. A thin layer of positive photoresist AZ 1505 (MicroChemicals) was deposited by spin-coating on the silicon wafer at 500 rpm for 10 s then 5000 rpm for 30 s, following by baking at 100 °C for 30 s. Then the wafer was patterned by direct-write lithography (DWL 66FS, Heidelberg Instruments Gmbh). After developing the photoresist by immersing the wafer in the metal ion free developer AZ 726 (MicroChemicals) for 45 s, the lithographic pattern is transferred onto the oxide layer by etching the silicon in buffered hydrofluoric acid. The photoresist film is no longer needed and therefore removed with acetone. In order to obtain groove silicon samples, the pre-patterned silicon wafers were submersed in 8% TMAH at 80 °C for 60 min to obtain slope-shaped grooves and 90 min to obtain V-shaped grooves.

### Surface characterization

Surface characterization was carried out by SEM using a FEI Quanta 600 environmental scanning electron microscope (Hillsboro, OR, USA) operating at accelerating voltages between 15 and 25 keV.

### Surface functionalization

Surface functionalization was carried out in a similar manner as described before [38]. The grooved silicon substrates were oxidized at 600 °C for 15 min. Then, the samples were treated in KOH (0.1 M) for 3 min and HNO_3_ (0.1 M) for 10 min to increase the density of surface hydroxy groups [39]. For collagen treatment of the substrates, the surface was chemically modified following the APTES–GTA–collagen sequence. In brief, oxidized samples were hydroxylated and silanized with APTES (Gelest) by exposure to a 10% (v/v) solution in anhydrous toluene for 2 h at room temperature, then washed in succession with toluene, ethanol and deionized water and, dried under a nitrogen flow. Afterwards, the samples were thermally cured at 120 °C overnight. The reaction with GTA was performed by exposure to a 10% (v/v) solution in anhydrous ethanol (Electron Microscopy Sciences) for 1 h at room temperature. The samples were rinsed in ethanol, deionized water and dried with nitrogen. Finally, the samples were incubated with collagen from bovine Achilles tendon (Lyophilized, Sigma-Aldrich) in a 10 mg/mL solution in phosphate-buffered saline (PBS; pH 7.4) at 4 °C overnight. The substrate was thoroughly washed with PBS and dried with a nitrogen flow.

### Cell seeding and culture

As described before [32–33], HAECs were purchased from Cascade Biologics TM (Portland, USA) and at the 5th passage were thawed and seeded on NunclonTM surface 24-well plates in the presence or absence (control conditions) of sterilized silicon substrates, at a density of approximately 1.9 × 10^4^ viable cells/mL and 4 × 10^3^ of viable cells/cm^2^. Throughout the experiment, cells were maintained in M200 medium supplemented with 2% (v/v) low serum growth supplement, 10 mg/mL gentamicin, 0.25 mg/mL amphotericin B (all from Life Technologies; Paisley, UK), 100 U/mL penicillin and 100 mg/mL of streptomycin (Laclinics, Spain). Cells were incubated at 37 °C in a humidified incubator (Heracell 150; Madrid, Spain) with an atmosphere containing 5% CO_2_.

### Cell viability and cytotoxicity

Cell viability was assessed by morphology using phase-contrast microscopy and by trypan blue dye exclusion test (Merck). Viability 97% was required for the thawed HAECs in order to guarantee the viability of the cells before starting each set of experiments.

The extent of cytotoxicity in each experimental condition was determined by a colorimetric assay that measures lactate dehydrogenase (LDH) activity (The LDH Cytotoxicity Detection Kit; Roche Applied Science, Germany). LDH is an intracellular enzyme that is released to the extracellular media when the cellular membrane is compromised as a result of adverse conditions. In the present work LDH activity was measured in cell-free culture supernatants collected after 24 h, 2 days, 3 days, 6 days and 7 days of incubating the cells on silicon substrates. A blank control (cells seeded in multi-well plate in the absence of silicon surface) was used as calibrator in all the experiments. Blank control values were set at 100% and the other conditions were calculated in relation to this reference value.

### Scanning electron microscopy (SEM)

HAECs were cultured on the functionalized silicon substrates for 2 days. After cell culture experiments, culture media were removed and cells were washed twice with PBS at 37 °C and afterwards fixed as previously described [31]. Afterwards, adhesion to silicon substrates, morphology and proliferation of HAECs were assessed using SEM (JEOL model JSM-6400), as described further below.

### Staining on actin and nuclei and fluorescence confocal microscopy

HAECs were cultured on the functionalized substrates for 2 and 7 days. After cell culture experiments, culture media were removed and cells were washed twice with PBS at 37 °C and afterwards fixed as previously described [31]. Actin-stain 670 phalloidin (Tebu-Bio) was used to stain the actin filaments of cytoskeleton (200 nM, 30 min), while NucGreen Dead 488 (Life Technologies) was used to stain the nuclei (2 drops/mL, 10 min). The fluorescence images were acquired using a Nikon Eclipse TE2000-E inverted microscope, equipped with a C1 laser confocal system (EZ-C1 software, Nikon). A 633 nm and 488 nm argon laser light was used as excitation sources for Phalloidin and NucGreen, respectively. Actin filaments and nuclei stain visualization using confocal microscopy was used to assess cellular morphology and adhesion, as described below.

### Cell behaviour assessment: adhesion, morphology and proliferation

Cell adhesion to substrates was assessed by quantifying the number of cells attached to such structures. Cell morphology was defined as the combination of circularity, alignment to the substrate structures and presence of filopodia. Circularity was calculated as the ratio between the minimum and maximum diameters. Values range from 0 to 1, where 0 represents an elongated cell and 1 a perfect circular shape. Alignment and presence of filopodia was estimated by visual assessment. Cell proliferation was calculated as the ratio of cell number at day 7 minus cell number at day 2.

### Statistical analyses

One-way analysis of variance (ANOVA) with Bonferroni and Dunnett post-hoc tests were used for multiple comparisons. A value of *p* < 0.05 was considered statistically significant. A prerequisite for the analytical quality of the model was the control of several aspects involved in the cellular process and analytical performance of measurements. Thus, the precision of the model was evaluated by calculating the standard deviation (SD), the standard error of the mean (SEM) and the coefficients of variation (CV) of the variables. All the results were analysed with the Statistical Package for the Social Sciences (SPSS) software (version 23.0).
